# Structural dataset for the fast-exchanging KRAS G13D

**DOI:** 10.1016/j.dib.2015.10.001

**Published:** 2015-10-17

**Authors:** Jia Lu, John Hunter, Anuj Manandhar, Deepak Gurbani, Kenneth D. Westover

**Affiliations:** University of Texas, Southwestern Medical Center, United States

**Keywords:** KRAS, GTPase, Cancer, X-ray crystallography

## Abstract

Cancers bearing the KRAS G13D mutation are notable for their distinct clinical behavior relative to other oncogenic KRAS mutations. We hypothesized that primary biochemical or biophysical properties of KRAS G13D might contribute to these clinical observations and as part of our study undertook structural studies using x-ray crystallography. In this data article we discuss several x-ray diffraction datasets that yielded structures of oncogenic KRAS mutants including a high resolution (1.13 Å) structure of KRAS G13D. The datasets are typical for high resolution x-ray diffraction data and allow the construction of atomic resolution, three dimensional structural models with high confidence. This data can be correlated with biochemical information such as defects in substrate binding kinetics, GTPase activities and interactions with the RAS effector RAF kinase.

**Specifications Table**

**Table:** 

Subject area	*Biology*
More specific subject area	*Structural Biology, Biochemistry*
Type of data	*Images, binary files, text files*
How data was acquired	*Advanced Photon Source beamline 19-ID in Chicago IL*
Data format	*PDB format text file. Binary structure factor file in mtz format. Analyzed by HKL2000/3000, Phenix and coot, Pymol, PDB*2*PQR with PARSE and PROPKA*
Experimental factors	*None*
Experimental features	*KRAS protein was crystallized by hanging drop vapor diffusion, cooled to 100 K and subjected to synchrotron radiation from which diffraction images were collected. Images were computationally analyzed to index reflections and extract associated intensities. Molecular replacement was used to obtain phase information. Computer aided modeling was used to generate refined atomic coordinates for KRAS proteins and to evaluate electrostatic potential*
Data source location	*Dallas, United States*
Data accessibility	*Protein coordinate data is publically available in the RCSB Protein Database*(http://www.rcsb.org/)*using the identifiers 4TQ9, 4QL3, 4TQA, 4WA7. X-ray diffraction images are available from the SBGRID data repository at*https://data.sbgrid.org/dataset/157/; doi:10.15785/SBGRID/157

**Value of the data**

• G13D has the most rapid nucleotide exchange kinetics among common KRAS mutants enabling hyper-auto-activation. A high resolution structure of G13D provides an explanation based on the electrostatic potential of the protein surface.
• KRAS structures may assist with efforts to design compounds that directly inhibit oncogenic KRAS proteins.
• KRAS structures may assist with constructing subclassification schemes for KRAS-containing tumors to direct tailored therapies.

## 1 Data

### 1.1 Data collection and structure determination of KRAS G13D

**Fig. 1:**
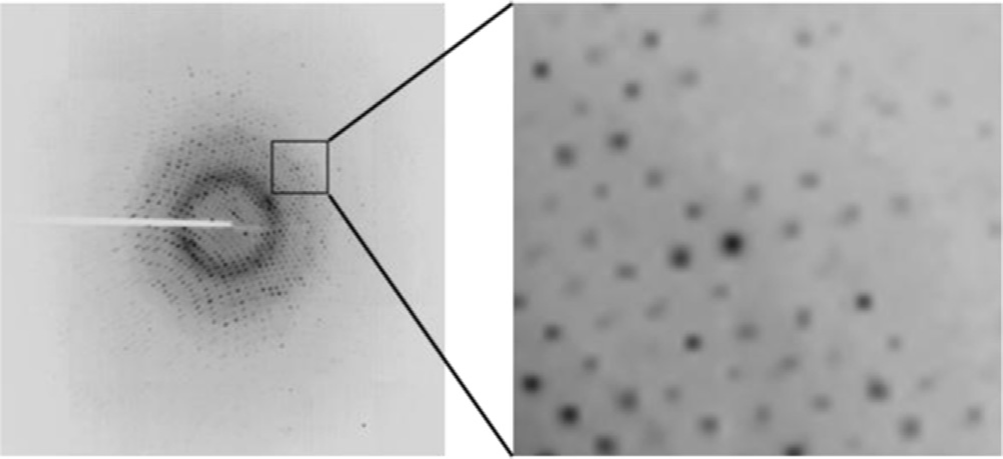
X-ray diffraction image from the G13D dataset collected at APS 19-1D.

The x-ray diffraction data set for KRAS G13D is similar in nature and quality to datasets for other KRAS mutants collected as part of this study. The primary KRAS G13D image data consists of 450 diffraction images that were each collected during a 0.5° rotation of the crystal for a total of 225° of diffraction data. The diffraction spots (reflections) are clean sharp and demonstrate a single lattice. There is no evidence of salt or crystalline water (ice) formation (Fig. 1). The KRAS G13D crystals were observed to belong to the monoclinic space group C2 with unit cell dimensions *a*=66.2 Å, *b*=41.3 Å, *c*=91.8 Å, *α*=*γ*=90° and *β*=105°. Spots with sufficient intensity above background to be statistically useful extended to a resolution of 1.13 Å with 99.7% of expected spots visible at that resolution. In total 111,363 unique reflections were measured with an overall redundancy of 4.4. All statistics for data processing indicate a high quality dataset (Table 1). Molecular replacement was used to obtain phase information and after several rounds of a combination of automated and manual refinements, a model consisting of 3188 non-hydrogen atoms was constructed with excellent statistics of 14% R-work, 16.9% R-free and 18.0 Å^2^ average B-factor. Given the large number of unique reflections and high resolution of the data, anisotropic B factor refinement was done. Table 1 summarizes the final statistics for data processing and refinement.

### 1.2 Crystal structure of KRAS G13D

#### 1.2.1 Electron density map

**Fig. 2:**
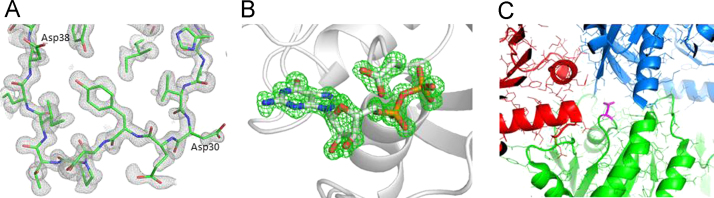
Representative portion of the 1.13 Å electron density map with the superimposed molecular model. (A) Atoms for switch I residues 30–38 are clearly observed in the 2*fo*-*fc* (*σ*=1.0) density map. (B) An *fo*-*fc* omit map (*σ*=3.0) for GDP and Asp13 side chain shows strong positive density for GDP and the Asp13 side chain. (C) Interface between symmetric molecules. Asp13 (showed in magenta) does not participate in any crystallographic contacts. Molecules in the crystal lattice are differentially colored red, green and blue.

**Fig. 3:**
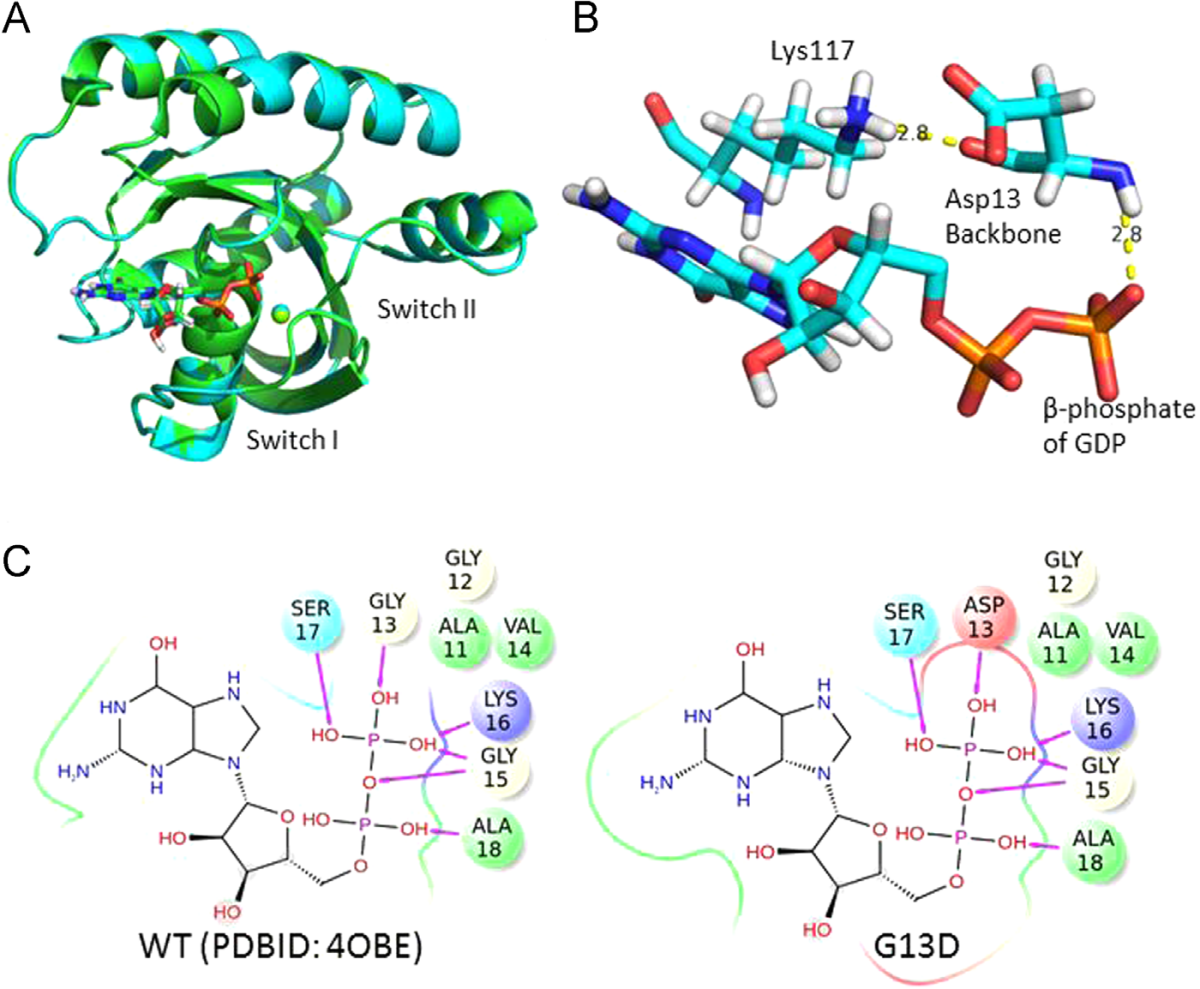
Crystal structures of KRAS WT (green) and G13D (cyan). (A) Superimposition of wild type KRAS and G13D. The conformations of switch I and II regions are almost identical. (B) Backbone interactions of G13D. The Asp13 main chain amide is 2.8 Å away from β-phosphate of GDP and carbonyl is 2.8 Å away from the amine of Lys117. (C) 2D scheme of Hydrogen bonding between β-phosphate of GDP and backbone of P-loop (residues 13–17) in WT (left) and G13D (right).

In this 1.13 Å structure, there are two molecules in the asymmetric unit and each shows strong and easily interpretable electron density for the backbone, as well as side chains (Fig. 2A). All loops, including switch I (residues 30–38) and II (residues 60–76) whose conformations undergo a conformational “switch” depending on if bound to GTP vs. GDP, are in well-defined density. Calculation of a *fo*-*fc* map where GDP and the side chain of residue 13 were omitted shows strong density corresponding to the aspartate side chain and GDP (Fig. 2B). The conformation of Asp13 is not constrained by intermolecular interactions in the crystal packing (Fig. 2C). We also identified strong electron density (14.0 sigma 2*fo-fc* map, 51.5 sigma *fo-fc* map) near the β-phosphate of GDP consistent with a coordinated Mg^2+^ atom as observed in prior KRAS structures. The G13D mutation has little effect on the overall structure of the protein with global RMSD of 0.146 Å compared with wildtype KRAS (PDBID 4OBE). Both switch I and II regions are in the inactive open conformation, similar to the wildtype and mutant KRAS structures we reported previously [1] (Fig. 3A).

#### 1.2.2 Guanine nucleotide binding site

Interactions between KRAS G13D and GDP are similar to previously reported interactions seen in wild type KRAS protein [2], [3]. Specifically the backbone nitrogen of residue 13 interacts with the β-phosphate of GDP, and the main chain carbonyl is hydrogen bound to the amine of Lys117. Introduction of Asp13 does not alter the position of its backbone, nor disrupt these interactions (Fig. 3B). In fact, the positioning of main chain atoms in the entire phosphate binding loop (also known as the P-loop, residues 13–17) are unaffected by this mutation, and remains within hydrogen bonding distance of the β-phosphate of GDP, stabilizing the negative charged β phosphate [4] (Fig. 3C). The aspartate side chain is positioned above the α-phosphate and is rotated towards the ribose ring of GDP with the carboxylic oxygen sitting ~3 angstroms from carbon-5 of the ribose sugar, although no direct interaction is observed. Tyr32 on switch I coordinates with a water molecule adjacent to the β-phosphate, allowing it to stabilize negative charges during GTP hydrolysis [5]. Modeling G13D onto the activated KRAS structure demonstrates that the side chain atoms of Asp13 are ~4 Å from Tyr32 and face the opposite side of the P-loop, and would not be predicted to have an impact on the interaction between Tyr32 and GTP.

### 1.3 Electrostatic potential maps of protein surface

**Fig. 4:**
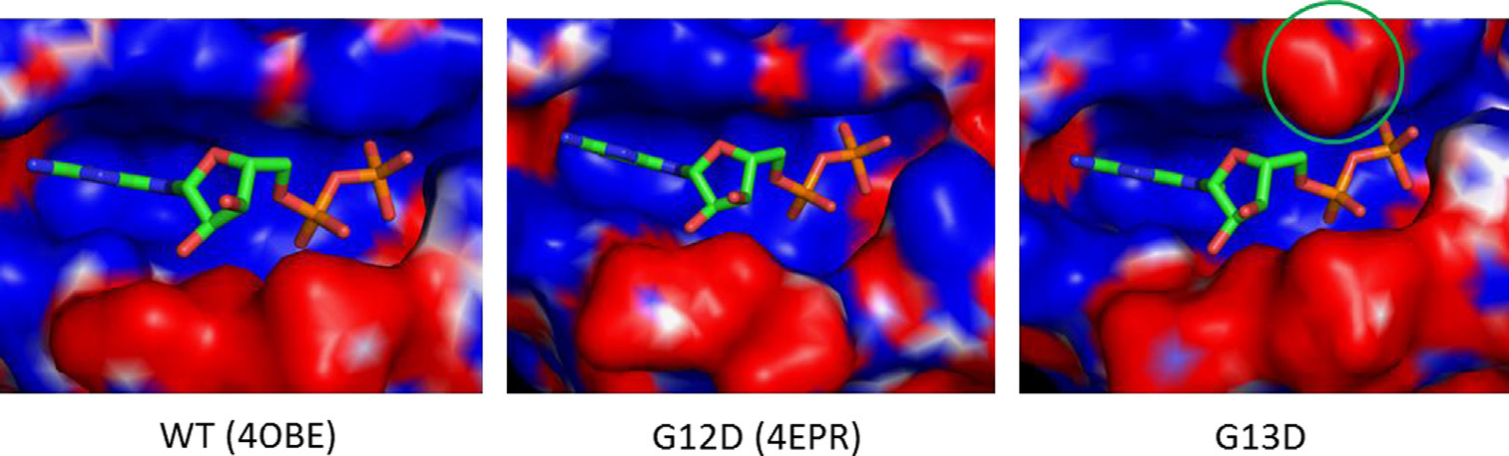
Electrostatic potential maps were calculated using the PDB2PQR server and APBS tools for WT, G12D and G13D. Positive charge is showed in blue and negative charge is showed in red. Negative charge from G13D are highlighted with a green circle.

As part of our study we measured the rate of nucleotide exchange of various common KRAS mutant isoforms and found that KRAS G13D stood out as having particularly high rates of nucleotide exchange (see Ref. [6], Fig. 1). In an attempt to understand this phenomenon we calculated the electrostatic potentials at the solvent interfaces of G13D, G12D and wild type KRAS. The P-loop region, which includes residues 12 and 13, is above the guanosine diphosphate and strongly positive facilitating binding of the negatively charged phosphates in wild type KRAS (Fig. 2, PDB ID: 4OBE). However, the carboxyl portion of aspartate 13 introduces a significant negative charge in this region, altering the local electrostatic environment. In contrast, the electrostatic map of G12D KRAS calculated from the published structure (PDB ID: 4EPR) demonstrates much less disruption of the positively charged binding pocket because in that case the side chain of Asp 12 is rotated away from the phosphates (Fig. 4). The repulsion between the negatively charged carboxylic acid of Asp13 and the α-phosphate of GTP or GDP effectively causes a more rapid exchange of guanine nucleotides as compared to wildtype KRAS and other KRAS mutant isoforms.

## 2 Experimental design, materials and methods

### 2.1 Protein preparation and crystallization

Protein was expressed and purified as described previously [1]. Point mutations were generated using the GeneArt® site-directed mutagenesis system (Life Technologies). Due to precipitation issue, 5 mM GDP was added to the buffer after IMAC column to stabilize the protein. Finally, G13D was buffer exchanged into 20 mM Tris pH 8.0, 50 mM NaCl, 1 mM DTT, 5 mM GDP and stored at −80 °C until use. KRAS G13D was unique amongst the KRAS mutants we purified in its requirement for GDP in the purification buffers to prevent protein precipitation. Crystals of KRAS mutants grew from hanging vapor diffusion drops with following condition: 0.2 M sodium acetate pH 4.5, 0.1 M Tris pH 8.5, 26% PEG 3350. 100–300 µM square cubic crystals normally appeared after 5 days incubation at 20 °C. Crystals were cryoprotected in 15% glycerol and flash frozen in liquid nitrogen.

### 2.2 Crystal structure determination

Diffraction images were collected at the advanced photon source beamline 19-ID. Data was integrated and scaled using HKL2000/3000 packages [7]. Molecular replacement was performed with 4OBE as the search model using Phaser software. Manual and automated model building and refinement were performed using Phenix package and coot software [8], [9]. Figure images were prepared using Pymol (The PyMOL Molecular Graphics System, Version 1.5.0.4 Schrödinger, LLC). Final model and scaled reflection data was deposited at the protein databank. Final collection and refinement statistics are presented in Table 1. 2D interaction schemes were prepared in Maestro (Schrödinger Release 2015-3: Maestro, version 10.3, Schrödinger, LLC, New York, NY, 2015). Electrostatic maps of WT KRAS (PDB ID 4OBE) and KRAS G13D were generated using the PDB2PQR server [10], [11] with the PARSE force field and PROPKA to assign protonation states. Images were prepared using the Pymol APBS tools plugin [12].

## Figures and Tables

**Table 1:** X-ray crystallography statistics for data collection and refinement.

**Data Collection**		**Refinement**	
Source	APS 19-1D	Resolution	27-1.13
Wavelength (Å)	0.97924	Reflections used	109,383
Space group	C2	Reflections for R-Free	1980
Unit cell		Non-hydrogen atoms	3188
*a*, *b*, *c* (Å)	66.2, 41.3, 114.5	Protein	2764
*α*, *β*, *γ* (deg.)	90, 105, 90	Water	424
Resolution (Å)	27.2-1.13	R-work	0.14
Unique reflections	111,363	R-free	0.169
Redundancy	4.4 (4.0)	RMS deviations	
Completeness (%)	99.7 (99.3)	Bond lengths (Å)	0.011
R-merge	0.05 (0.52)	Bond angles (°)	1.476
<I/*σ*>	34.6 (2.0)	Average B-factor (Å^2^)	18
Wilson B-factor (Å^2^)	11.7		
		Ramachandran plot (%) Favored/allowed/disallowed	99.7/0.3/0.0
		MolProbity score	0.74 (99%)

## References

[1] Hunter J.C., Gurbani D., Ficarro S.B., Carrasco M.A., Lim S.M., Choi H.G., Xie T., Marto J.A., Chen Z., Gray N.S., Westover K.D. (2014). in situ selectivity profiling and crystal structure of SML-8-73-1, an active site inhibitor of oncogenic K-Ras G12C. Proc. Natl. Acad. Sci. USA.

[2] Pai E.F., Kabsch W., Krengel U., Holmes K.C., John J., Wittinghofer A. (1989). Structure of the guanine-nucleotide-binding domain of the Ha-ras oncogene product p21 in the triphosphate conformation. Nature.

[3] Milburn M.V., Tong L., deVos A.M., Brunger A., Yamaizumi Z., Nishimura S., Kim S.H. (1990). Molecular switch for signal transduction: structural differences between active and inactive forms of protooncogenic ras proteins. Science.

[4] Du X., Sprang S.R. (2009). Transition state structures and the roles of catalytic residues in GAP-facilitated GTPase of Ras as elucidated by (18)O kinetic isotope effects. Biochemistry.

[5] Buhrman G., Holzapfel G., Fetics S., Mattos C. (2010). Allosteric modulation of Ras positions Q61 for a direct role in catalysis. Proc. Natl. Acad. Sci. USA.

[6] Hunter J.C., Manandhar A., Carrasco M.A., Gurbani D., Gondi S., Westover K.D. (2015). Biochemical and structural analysis of common cancer-associated KRAS mutations. Mol. Cancer Res..

[7] Otwinowski Z., Minor W. (1997). Processing of X-ray diffraction data collected in oscillation mode. Method Enzymol..

[8] Adams P.D., Afonine P.V., Bunkoczi G., Chen V.B., Davis I.W., Echols N., Headd J.J., Hung L.W., Kapral G.J., Grosse-Kunstleve R.W., McCoy A.J., Moriarty N.W., Oeffner R., Read R.J., Richardson D.C., Richardson J.S., Terwilliger T.C., Zwart P.H. (2010). PHENIX: a comprehensive Python-based system for macromolecular structure solution. Acta Crystallogr. Sect. D Biol. Crystallogr..

[9] Emsley P., Lohkamp B., Scott W.G., Cowtan K. (2010). Features and development of Coot. Acta Crystallogr. Sect. D Biol. Crystallogr..

[10] Dolinsky T.J., Czodrowski P., Li H., Nielsen J.E., Jensen J.H., Klebe G., Baker N.A. (2007). PDB2PQR: expanding and upgrading automated preparation of biomolecular structures for molecular simulations. Nucleic Acids Res..

[11] Dolinsky T.J., Nielsen J.E., McCammon J.A., Baker N.A. (2004). PDB2PQR: an automated pipeline for the setup of Poisson–Boltzmann electrostatics calculations. Nucleic Acids Res..

[12] Baker N.A., Sept D., Joseph S., Holst M.J., McCammon J.A. (2001). Electrostatics of nanosystems: application to microtubules and the ribosome. Proc. Natl. Acad. Sci. USA.

